# Engineered Biosynthesis of Regioselectively Modified Aromatic Polyketides Using Bimodular Polyketide Synthases

**DOI:** 10.1371/journal.pbio.0020031

**Published:** 2004-02-17

**Authors:** Yi Tang, Taek Soon Lee, Chaitan Khosla

**Affiliations:** **1**Department of Chemical Engineering, Stanford UniversityStanford, CaliforniaUnited States of America; **2**Department of Chemistry, Stanford UniversityStanford, CaliforniaUnited States of America; **3**Department of Biochemistry, Stanford UniversityStanford, CaliforniaUnited States of America

## Abstract

Bacterial aromatic polyketides such as tetracycline and doxorubicin are a medicinally important class of natural products produced as secondary metabolites by actinomyces bacteria. Their backbones are derived from malonyl-CoA units by polyketide synthases (PKSs). The nascent polyketide chain is synthesized by the minimal PKS, a module consisting of four dissociated enzymes. Although the biosynthesis of most aromatic polyketide backbones is initiated through decarboxylation of a malonyl building block (which results in an acetate group), some polyketides, such as the estrogen receptor antagonist R1128, are derived from nonacetate primers. Understanding the mechanism of nonacetate priming can lead to biosynthesis of novel polyketides that have improved pharmacological properties. Recent biochemical analysis has shown that nonacetate priming is the result of stepwise activity of two dissociated PKS modules with orthogonal molecular recognition features. In these PKSs, an initiation module that synthesizes a starter unit is present in addition to the minimal PKS module. Here we describe a general method for the engineered biosynthesis of regioselectively modified aromatic polyketides. When coexpressed with the R1128 initiation module, the actinorhodin minimal PKS produced novel hexaketides with propionyl and isobutyryl primer units. Analogous octaketides could be synthesized by combining the tetracenomycin minimal PKS with the R1128 initiation module. Tailoring enzymes such as ketoreductases and cyclases were able to process the unnatural polyketides efficiently. Based upon these findings, hybrid PKSs were engineered to synthesize new anthraquinone antibiotics with predictable functional group modifications. Our results demonstrate that (i) bimodular aromatic PKSs present a general mechanism for priming aromatic polyketide backbones with nonacetate precursors; (ii) the minimal PKS controls polyketide chain length by counting the number of atoms incorporated into the backbone rather than the number of elongation cycles; and (iii) in contrast, auxiliary PKS enzymes such as ketoreductases, aromatases, and cyclases recognize specific functional groups in the backbone rather than overall chain length. Among the anthracyclines engineered in this study were compounds with (i) more superior activity than R1128 against the breast cancer cell line MCF-7 and (ii) inhibitory activity against glucose-6-phosphate translocase, an attractive target for the treatment of Type II diabetes.

## Abstract 2

Analogues of microbial secondary metabolites, which include many antibiotics and antitumor drugs, can be engineered from unusual primer units of the polyketide backbone to create new medicinal compounds with promising novel pharmacological properties

## Introduction

Polyketides are a large class of structurally and pharmacologically diverse molecules, including many antibiotics and antitumor drugs (O'Hagan 1991). They are produced as secondary metabolites primarily by bacteria and fungi (Hopwood 1997). Analogous to fatty acid synthases (FASs), polyketide synthases (PKSs) catalyze the biosynthesis of polyketides through repetitive C–C bond-forming reactions between selected acyl-CoA-derived building blocks (Cane et al. 1998). However, in contrast to fatty acid biosynthesis, the carbon chain backbones of polyketides exhibit greater variety with respect to the choice of acyl-CoA building blocks and the degree of reduction of β-ketone functional groups that result after each round of chain elongation.

**Figure 1:**
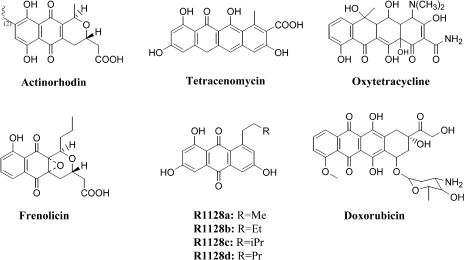
Examples of Aromatic Polyketides Actinorhodin (S. coelicolor) and tetracenomycin (S. glaucescens) are primed by acetate groups through decarboxylation of malonyl-CoA. Actinorhodin and tetracenomycin are synthesized by the *act* and *tcm* PKSs, respectively. Oxytetracycline (S. rimosus), frenolicin (*S. roseofulvus)*, R1128 (*S.* R1128), and doxorubicin (S. peucetius) are primed by nonacetate units as shown. Notably, R1128 family of compounds (a–d) are primed with different alkyl units. Oxytetracycline, frenolicin, R1128, and daunorubicin are synthesized by the *otc*, *frn*, R1128, and *dxr* PKSs, respectively. Actinorhodin and tetracenomycin represent much-studied models of aromatic polyketide biosynthesis. Oxytetracycline is a commonly prescribed antibiotic. Frenolicin is a potent antiparasitic agent. R1128 is an estrogen receptor antagonist that shows minimal agonist activity. Doxorubicin is a widely used anticancer drug in treating late-stage tumors.

**Figure 2:**
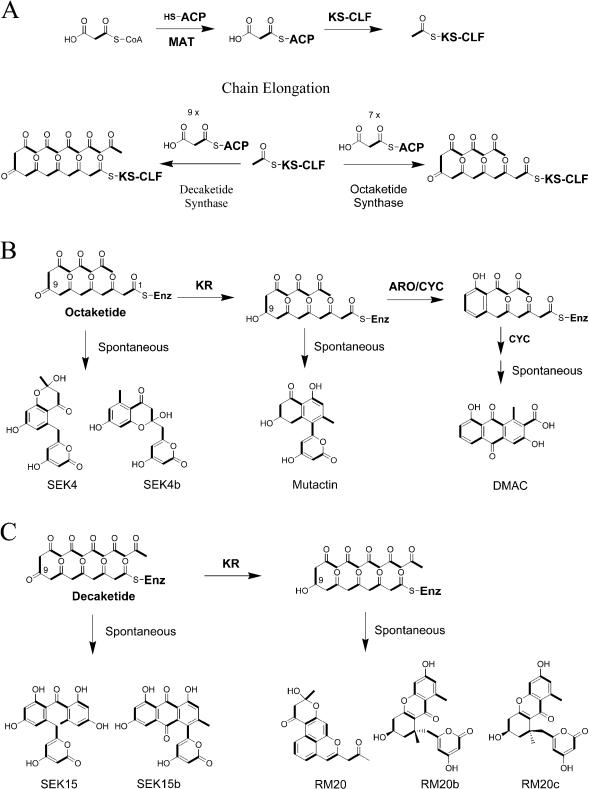
Biosynthesis of Acetate-Primed Polyketides (A) Minimal PKS is necessary and sufficient for the synthesis of a complete polyketide chain. KS-CLF is the condensing enzyme in the minimal PKS, catalyzing each round of condensation between malonyl-ACP and the growing polyketide chain. ACP serves as the carrier for malonyl units, and it is malonylated by the MAT associated with FAS. Chain synthesis initiates with the decarboxylation of malonyl-ACP to acetyl-ACP by the KS-CLF for most aromatic PKSs. The acetyl unit is then transferred to the KS-CLF and primes the enzyme for subsequent condensations. The overall chain length is controlled by the KS-CLF complex. An octaketide synthase (e.g., *act* PKS) uses a total of eight malonyl equivalents (including the primer), while a decaketide synthase (e.g., *tcm* PKS) uses a total of ten malonyl equivalents. (B) An octaketide can spontaneously form SEK4 and SEK4b in the absence of tailoring enzymes. Members of the *act* KR family can regioselectively reduce the octaketide at C-9, which can then spontaneously form mutactin in the absence of AROs and CYCs. When bifunctional ARO/CYC (e.g., *act*VII) and second-ring CYC (e.g., *act*IV) are present, the reduced octaketide can be transformed into the anthraquinone DMAC. (C) A decaketide can spontaneously form SEK15 and SEK15b in the absence of tailoring enzymes. KR can regioselectively reduce the C-9 carbonyl. A reduced decaketide can spontaneously form RM20, RM20b, and RM20c. Not shown are the other tailoring enzymes associated with decaketides, which can transform the nascent decaketide into tetracycline or anthracycline structures.

In complex polyketides, such as the macrolide erythromycin, biosynthetic variability arises from independent control of each round of chain elongation by one module of enzymes within a multimodular PKS (Cane et al. 1998). (The term *module* used in this report refers to a collection of dissociated enzymes. The elongation module consists of enzymes involved in chain extension steps of polyketide biosynthesis, while the initiation module consists of enzymes involved in the nonacetate priming of certain aromatic PKSs.) However, the polyketide backbones of most bacterial aromatic polyketides (Figure 1) are synthesized by a single dissociated enzymatic module comprised of a heterodimeric ketosynthase–chain length factor (KS-CLF) complex that catalyzes chain initiation and iterative elongation, an acyl-carrier protein (ACP) that shuttles malonyl extender units to the active site of KS-CLF as malonyl-S-ACP intermediates, and a malonyl-CoA:ACP acyl transferase (MAT), which catalyzes acyl transfer between malonyl-CoA and the ACP and is shared between the PKS and the housekeeping FAS(s) (Revill et al. 1995; Khosla et al. 1999). For example, the minimal PKS from the actinorhodin (*act*) biosynthetic pathway synthesizes an octaketide (C_16_) backbone from eight malonyl-CoA equivalents, whereas the tetracenomycin (*tcm*) minimal PKS synthesizes a decaketide (C_20_) backbone from ten equivalents of malonyl-CoA (Figure 2) Although a large number of “unnatural” natural products have been engineered to date by genetic manipulation of bacterial aromatic PKSs (Khosla and Zawada 1996; Rawlings 1999; Shen et al. 1999), most of this variety has resulted from the combinatorial manipulation of ketoreductases (KRs), aromatases (AROs), and cyclases (CYCs) that ordinarily interact with minimal PKS subunits to channel the exceptionally high reactivity of a poly-β-ketone intermediate (Figure 2) into the observed natural product (McDaniel et al. 1995). The development of generally applicable methods for chemo- and regioselective modification of natural and unnatural bacterial aromatic polyketides is an important goal for the medicinal chemist and, more recently, the biosynthetic engineer.

**Figure 3:**
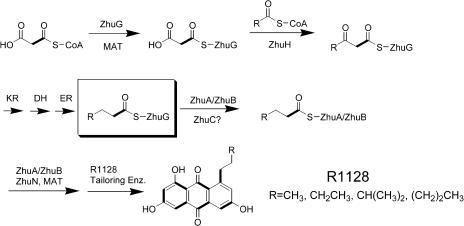
Proposed Priming Mechanisms for R1128 PKS An independent loading module consisting of ZhuG, ZhuH, and ZhuC can generate an alkylacyl-ZhuG intermediate (boxed) from malonyl-CoA and short chain acyl-CoAs such as propionyl-CoA and isobutyryl-CoA. The precursor selectivity is determined by the KSIII analog ZhuH. Ketoreductase, dehydratase, and enoylreductase associated with FAS are presumed to transformed the β-ketoacyl-ZhuG moiety into alkylacyl-ZhuG. The alkylacyl-ZhuG is then able to prime the minimal PKS module (consisting of the ZhuB [KS], ZhuA [CLF], ACP [ZhuN], and MAT) and initiate polyketide synthesis. The mechanism by which the transacylation occurs is not known and is possibly catalyzed by unassigned, but essential, enzyme ZhuC. Homologs of ZhuG, ZhuH, and ZhuC are present in the *frn* PKS (FrnJ, FrnK, and FrnI, respectively) as well.

The primer unit of a polyketide backbone is an attractive site for introducing unnatural building blocks. For example, genetic and chemobiosynthetic approaches have been devised to replace the natural primer units in the polyketide backbones of erythromycin, avermectin, and rapamycin with a broad range of unnatural functional groups (Jacobsen et al. 1997; Marsden et al. 1998; Lowden et al. 2001; Long et al. 2002). However, most aromatic PKSs initiate polyketide biosynthesis through decarboxylation of malonyl-ACP, resulting in an invariant acetyl primer unit (Figure 2A) (Bisang et al. 1999; Dreier and Khosla 2000). Important antitumor antibiotics, such as the anthracycline doxorubicin, are primed with propionate units. Recently, we have investigated the biosynthesis of the estrogen receptor antagonist R1128 (Hori et al. 1993) and the antiparasitic agent frenolicin (Bibb et al. 1994), two aromatic polyketides that are apparently derived from nonacetate primer units (see Figure 1). These bimodular PKSs are comprised of a dissociated initiation module consisting of a homodimeric KS (ZhuH, named for R1128 PKS), an acyl transferase (AT) (ZhuC), and an ACP (ZhuG), and an elongation module consisting of a KS-CLF (ZhuA–ZhuB), a second ACP (ZhuN), and the MAT (borrowed from the housekeeping FAS). The proposed biosynthetic mechanism of the R1128 PKS is shown in Figure 3 (Marti et al. 2000). Biochemical studies have revealed that the KS subunits of the initiation and elongation modules have specific protein–protein interactions with ACPs from the same module (Tang et al. 2003), suggesting that it may be possible to functionally coexpress these initiation modules with heterologous minimal PKSs, so as to regioselectively incorporate nonacetate primer units into aromatic polyketides. Of particular interest is the R1128 initiation module, since it is known to have broad substrate specificity (Meadows and Khosla 2001) and the X-ray crystal structure of its KS subunit has been solved (Pan et al. 2002). Here we demonstrate the biosynthetic utility of the R1128 initiation module by synthesizing a variety of anthraquinone antibiotics, some with significant biological activities. In the course of these studies, fundamentally novel and unanticipated properties of bacterial aromatic PKSs have been elucidated.

## Results

Using the host/vector system first described by McDaniel et al. (1993a), several combinations of initiation modules, minimal PKSs, and auxiliary PKS subunits were coexpressed and analyzed. Streptomyces coelicolor CH999, which contains a chromosomal deletion of the entire *act* gene cluster, was used as the host strain, whereas the shuttle vector pRM5 was used as an expression plasmid. The polyketide product profiles of the recombinant strains are summarized in Table 1.

**Table 1:**
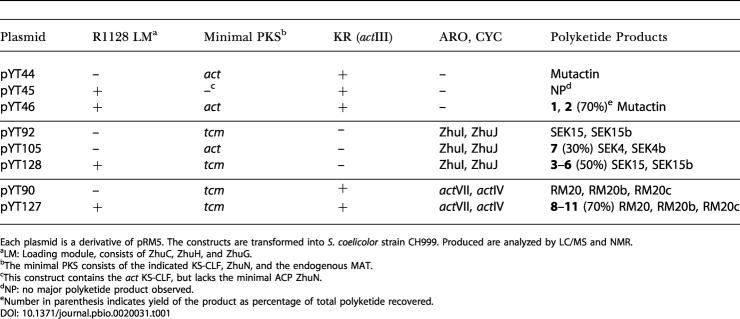
Plasmids Constructions and Resulting Polyketide Products

Each plasmid is a derivative of pRM5. The constructs are transformed into S. coelicolor strain CH999. Produced are analyzed by LC/MS and NMR

^a^LM: Loading module, consists of ZhuC, ZhuH, and ZhuG

^b^The minimal PKS consists of the indicated KS-CLF, ZhuN, and the endogenous MAT

^c^This construct contains the *act* KS-CLF, but lacks the minimal ACP ZhuN

^d^NP: no major polyketide product observed

^e^Number in parenthesis indicates yield of the product as percentage of total polyketide recovered

### Recombination of an Initiation Module and a Heterologous Minimal PKS

Guided by our recent observation that the KS–ACP pairs of initiation and elongation PKS modules have orthogonal molecular recognition features, we first attempted to coexpress the R1128 initiation module with the *act* minimal PKS. The *zhuC* (AT)*, zhuH* (KS), *and zhuG* (ACP) genes from the R1128 gene cluster were coexpressed with the genes encoding the *act* KS-CLF, ZhuN (ACP), and the *act* KR (on plasmid pYT46). Control plasmids pYT44, lacking the R1128 initiation module, and pYT45, lacking *zhuN*, were also constructed and characterized. All three plasmids were introduced into S. coelicolor CH999 by transformation, and polyketides products were analyzed by liquid chromatography/mass spectrometry (LC/MS) and nuclear magnetic resonance (NMR) spectroscopy.

**Figure 4:**
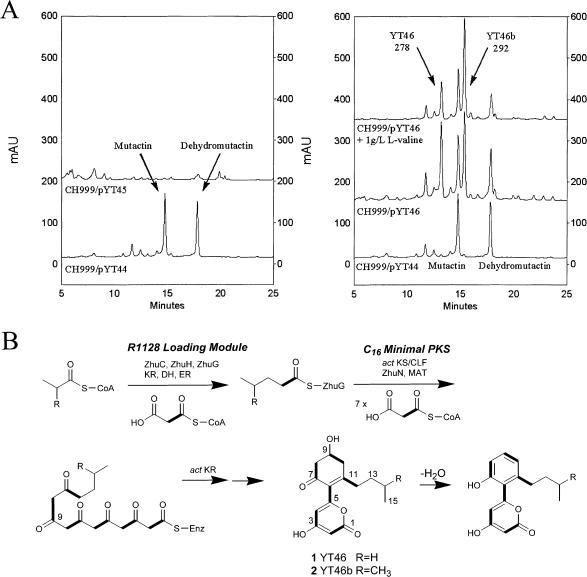
Engineered Biosynthesis of YT46 (1) and YT46b (2) (A) HPLC trace of extracts from CH999/pYT44-pYT46. A linear gradient of 20%–60% CH_3_CN in water was used. (Left) CH999/pYT44, which only has the minimal PKS module from *act* PKS and KR, produced the expected mutactin (and the dehydrated dehydromutactin). CH999/pYT45, which contains the R1128 loading module and an incomplete minimal PKS, produced no major polyketides. (Right) Upon coexpressing the R1128 loading module with a functional *act* minimal PKS (in the presence of KR), two new major polyketides were produced with the indicated masses. When 1 g/l of valine was added to the growth medium, the yield of the compound with mass of 292 doubled (traces not drawn to the same scale). The two compounds are identified as YT46 and YT46b. (B) Engineered biosynthesis of YT46 and YT46b. YT46 (1) is derived from propionate, while YT46b (2) is derived from isobutyrate. Alkylacyl-ZhuG supplied by the loading module is able to prime the *act* minimal PKS efficiently. Incorporation of the alkylacyl moiety by the KS-CLF led to a decrease in the number of extender units incorporated in the final chain. The octaketide synthase is now only able to synthesize an alkyl hexaketide. The KR is able to regioselectively reduce the alkyl-hexaketide at the expected C-9 position. The reduced hexaketide spontaneously form the novel bicyclic structure observed in 1 and 2. Dehydrated versions of 1 and 2 are also observed (outside of limits shown in [A]).

S. coelicolor CH999/pYT44 produced mutactin (and its dehydrated derivative, dehydromutactin), the expected products of the *act* minimal PKS in the presence of *act* KR (McDaniel et al. 1994a), whereas CH999/pYT45 did not produce any polyketide, consistent with the requirement for separate ACPs to support turnover of the initiation and elongation modules in a bimodular aromatic PKS (Figure 4A) (Tang et al. 2003). Remarkably, CH999/pYT46 produced two new polyketide products in addition to mutactin; these new polyketides were isolated with a combined yield of 40 mg/l, representing 70% of total polyketides produced by this host. The two compounds had molecular masses of 278 and 292. (The 14 mass unit difference is suggestive of one methylene unit difference between the two compounds.) Isotopic labeling studies indicated that the compounds were derived from six acetate equivalents. NMR analyses (Table 2) revealed that the two compounds, YT46 (**1**) and YT46b (**2**), had structures shown in Figure 4. YT46 and YT46b are identical, with the exception of a branched methyl group in YT46b. Each compound contains an α-pyrone moiety, which is commonly observed in aberrantly cyclized aromatic polyketides (Yu et al. 1998). The C-9 carbonyl groups were selectively reduced by the *act* KR. Supplementing the growth medium with^13^C-labeled sodium propionate revealed that the alkyl moiety of **1** was derived from propionyl-CoA. Similarly, supplementing the growth medium with 1 g/l of L-valine resulted in a 2-fold increase in the level of **2**, suggesting the branched alkyl group observed in **2** was derived from isobutyryl-CoA, a primary catabolite of L-valine (Zhang et al. 1999). These findings are consistent with in vitro characterization of the substrate specificity of ZhuH (Meadows and Khosla 2001). Among substrates tested, ZhuH had an 11-fold higher specificity for both propionyl-CoA and isobutyryl-CoA over the next best substrate, acetyl-CoA (Meadows and Khosla 2001). YT46 analogs generated by incorporation of isovaleryl and butyryl primer units were also detectable by LC/MS, although these compounds were present at lower levels. Dehydrated analogs of **1 **and **2 **were also observed in CH999/pYT46. (Purified **1 **and **2 **slowly dehydrate at room temperature.)

**Table 2:**
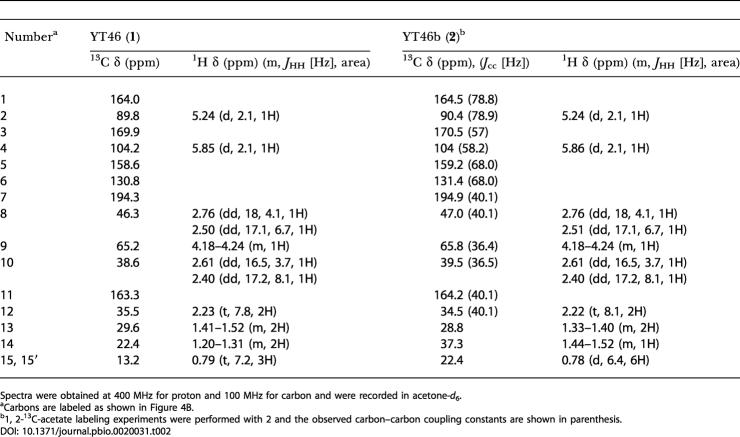
Proton and Carbon NMR Data for YT46 (**1**) and YT46b (**2**)

Spectra were obtained at 400 MHz for proton and 100 MHz for carbon and were recorded in acetone-*d*_6_

^a^Carbons are labeled as shown in Figure 4B

^b^1, 2-^13^C-acetate labeling experiments were performed with 2 and the observed carbon–carbon coupling constants are shown in parenthesis

The biosynthesis of **1 **and **2** by a recombinant bimodular PKS consisting of the *act* minimal PKS and the R1128 initiation module supported our hypothesis that nonacetate-primed polyketides could be biosynthesized by combinatorial expression of heterologous initiation and elongation PKS modules from bacterial aromatic PKSs. Indeed, the *act* KS-CLF, which is normally primed exclusively by acetate (generated via decarboxylation of a malonyl unit), has a remarkably strong preference for the diketide product of the R1128 initiation module. However, unexpectedly, the incorporation of a longer chain substrate into the catalytic cycle of the *act* minimal PKS results in a reduced number of malonyl units utilized during iterative chain extension (Figure 4B). Compound **1 **is a hexaketide whose backbone consists of 15 C atoms. Thus, upon introduction of an initiation PKS module into the overall catalytic cycle, the octaketide synthase retains its carbon chain length specificity, rather than executing the normal number of extension cycles. In contrast, the *act* KR retains its selectivity for the C-9 carbonyl in the nascent polyketide backbone, notwithstanding structural differences between a hexaketide and an octaketide.

### In Vivo Reconstitution of R1128 Biosynthesis Using a Heterologous Bimodular PKS

**Figure 5:**
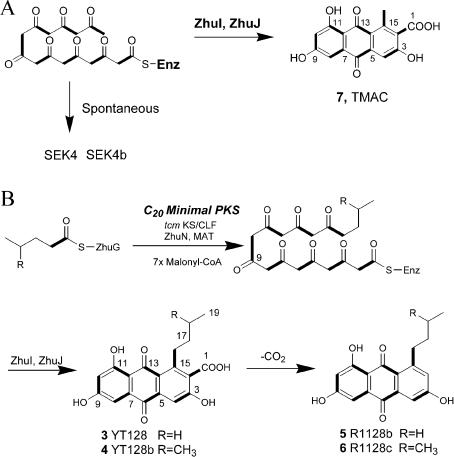
Engineered Biosynthesis of TMAC (7), YT128 (3), and YT128b (4) (A) ZhuI and ZhuJ are CYCs specific for unreduced octaketides. CH999/pYT105, which coexpressed ZhuI and ZhuJ from the R1128 PKS with the *act* minimal PKS, produced the anthraquinone compound TMAC (7). ZhuI and ZhuJ are thus able to cyclize unreduced octaketides. Previously characterized octaketide CYCs *act* ARO/CYC and *act* CYC are specific for reduced octaketides only. ZhuI and ZhuJ are used for reconstituting R1128 biosynthesis. (B) Reconstitution of R1128 biosynthesis using the heterologous combination of *tcm* minimal PKS, R1128 loading module, and CYCs ZhuI and ZhuJ. The alkylacyl-ZhuG intermediate synthesized by the loading module is able to prime the decaketide synthase from *tcm* minimal PKS. Owing to backbone size restrictions, the *tcm* KS-CLF primed with the alkylacyl groups are only able to extend the polyketide by seven additional malonyl units, resulting in an alkyl-octaketide. ZhuI and ZhuJ are able to transform the unreduced octaketide into YT128 (3) and YT128b (4). The decarboxylated versions of 3 and 4, which are R1128b (5) and R1128c (6), respectively, are also observed in the extracts of CH999/pYT128.

To validate the generality of the above observations, we sought to reconstitute the biosynthesis of R1128 family of compounds in S. coelicolor using a heterologous combination of initiation and elongation PKS modules (Figure 5). It follows from the above analysis that to synthesize an alkyl-primed anthraquinone such as **3** and **4**, the following catalytic components are needed: (i) an initiation module; (ii) a decaketide minimal PKS that can extend the five to six carbon products of the initiation module by seven more extender units to yield a 19–20 carbon polyketide; and (iii) appropriate ARO and CYC subunits.

The R1128 family of antibiotics represents a unique set of anthraquinones that contain an unreduced C-9 carbonyl (present as an enolic C-9 hydroxyl in R1128). Since members of the *act* ARO/CYC family are unable to cyclize an unreduced octaketide (McDaniel et al. 1994b) and since enzymes from the *tcm* ARO/CYC family have alternative regiospecificity of cyclization (McDaniel et al. 1995), AROs and CYCs were sought from the R1128 biosynthetic pathway. ZhuI and ZhuJ are two putative enzymes present in the R1128 PKS (Marti et al. 2000). ZhuI, which is homologous to the *act* ARO/CYC, was predicted to be a first ring CYC, while ZhuJ was predicted to be a second ring CYC. To test these hypotheses, plasmids pYT105 and pYT92 (see Table 1) were constructed, coexpressing ZhuI and ZhuJ with the *act* minimal PKS and the *tcm* minimal PKS, respectively. Analysis of compounds produced by CH999/pYT92 revealed the decaketides SEK15 and SEK15b as the major products, suggesting ZhuI and ZhuJ did not recognize an unreduced decaketide. However, the anthraquinone compound (Bartel et al. 1990), 3,6,8-trihydroxy-1-methylanthraquinone-2-carboxylic acid (TMAC) (**7**; also known as laccaic acid D, a well known plant-derived pigment) (Figure 5A; Table 2; atoms are numbered according to order in polyketide backbone), was isolated from CH999/pYT105 at 10 mg/l, in addition to the known products of *act* minimal PKS, SEK4 and SEK4b (20 mg/l). Thus, ZhuI and ZhuJ are able to cyclize an unreduced octaketide into the corresponding anthraquinone. The incomplete transformation of nascent octaketides into TMAC may be due to the fact that an acetate-primed octaketide is not a natural substrate of the two CYCs (see below).

The identification of ZhuI and ZhuJ as the appropriate CYCs for the synthesis of R1128-like anthraquinones prompted the design of pYT128, which coexpresses *tcm* KS-CLF, ZhuN, ZhuI, ZhuJ, and the R1128 initiation module. Plasmid pYT92 (which lacks the initiation module) was used as the negative control. In addition to the decaketides SEK15 and SEK15b, two new anthraquinone compounds, YT128 (**3**) and YT128b (**4**), were isolated at comparable levels (7 mg/l each) from S. coelicolor CH999/pYT128. The two compounds account for 50% of total polyketides produced by this recombinant strain. [^13^C]Propionate and valine feeding experiments verified the alkyl groups installed at C-16 were indeed derived from either propionyl-CoA or isobutyryl-CoA. NMR and MS analyses confirmed the identities of **3 **and **4 **as alkyl-primed TMAC analogs (Table 3). The natural products, R1128b (**5**) and R1128c (**6**) (i.e., decarboxylated derivatives of **3** and **4**), were present at an approximately 20% level to that of **3** and **4**.

**Table 3:**
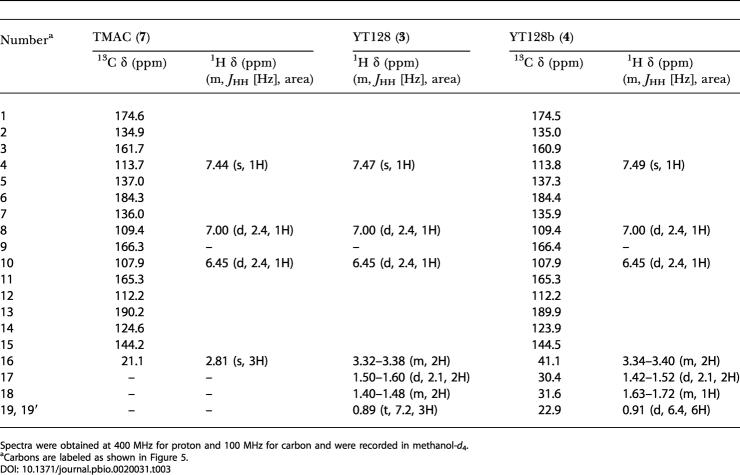
Proton and Carbon NMR Data for TMAC (**7)**, YT128 (**3**), and YT128b (**4**)

Spectra were obtained at 400 MHz for proton and 100 MHz for carbon and were recorded in methanol-*d*_4_

^a^Carbons are labeled as shown in Figure 5

The engineered biosynthesis of compounds **3**–**6** further validated our hypothesis that the initiation module of the R1128 PKS could productively interact with elongation modules from any bacterial aromatic PKS. Moreover, by incorporating a primer unit with at least five carbon atoms, the *tcm* KS-CLF effectively became an octaketide synthase with respect to the rest of the polyketide molecule. This was analogous to the conversion of the *act* KS-CLF into a hexaketide synthase in the presence of the R1128 initiation module. It also suggested that the R1128 KS-CLF was intrinsically a decaketide synthase. Finally, analogous to the *act* KR, ZhuI and ZhuJ were programmed to recognize full-length polyketide chains based upon the number of β-carbonyl groups, rather than the carbon chain length of the backbone. Our findings below suggest this is a general property for all CYCs. It should be noted that the CYCs ZhuI and ZhuJ were apparently more efficient in processing the unreduced alkyl-primed octaketide than an acetate-primed octaketide, since no alkyl-primed analogs of SEK4 and SEK4b were observed in extracts of CH999/pYT128. (In the absence of ZhuI and ZhuJ, alkyl-primed versions of both SEK4 and SEK4b are the major polyketides produced [data not shown]).

### Engineered Biosynthesis of Novel Anthraquinones Using Bimodular PKSs

**Figure 6:**
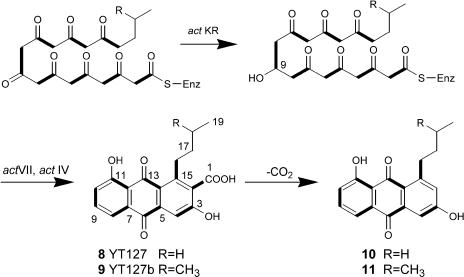
Engineered Biosynthesis of DMAC Analogs YT127 (8) and YT127b (9) When the bimodular PKS containing *tcm* minimal PKS and R1128 loading module are coexpressed with tailoring enzymes KR, *act* ARO/CYC, and *act* CYC, the desired compounds 8 and 9 were produced. The decarboxylated versions of 8 and 9 are 10 and 11, respectively. All three tailoring enzymes are able to process the unnatural alkyl-octaketide.

To demonstrate the utility of hybrid bimodular PKSs for the rational design of new analogs of known polyketides, we targeted the engineered biosynthesis of alkyl-primed 3,8-dihydroxy-methylanthraquinone carboxylic acid (DMAC) analogs **8** and **9** (Figure 6). Specifically, we inserted the *act* KR gene into pYT128, along with replacing *zhuI* and *zhuJ* with genes encoding *act* ARO and *act* CYC, to arrive at the plasmid pYT127. We rationalized that the *act* CYCs should be able to recognize the reduced, alkyl-primed octaketide. A control plasmid (pYT90) lacking the R1128 initiation module was also constructed, transformed, and analyzed.

In the absence of the initiation module, the *tcm* minimal PKS outfitted with the *act* KR produced the expected polyketides RM20, RM20b, and RM20c (McDaniel et al. 1993a). The targeted anthraquinone carboxylic acids **8** and **9** were isolated at high titers (15 mg/l each, 70% of total polyketide products) in CH999/pYT127. The identities of **8 **and **9 **were verified by NMR and MS (Table 4). Decarboxylated analogs of both compounds were also observed; these compounds are alkyl-primed analogs of the natural product aloesaponarin II. These findings confirmed that, analogous to ZhuI and ZhuJ, the *act* KR, *act* ARO, and *act* CYC were able to process the octaketide intermediate possessing unnatural functional groups at C-16. Thus, it appears that the substrate recognition features of all auxiliary PKS subunits have evolved to monitor the number of β-ketone functional groups present in the polyketide chain.

**Table 4:**
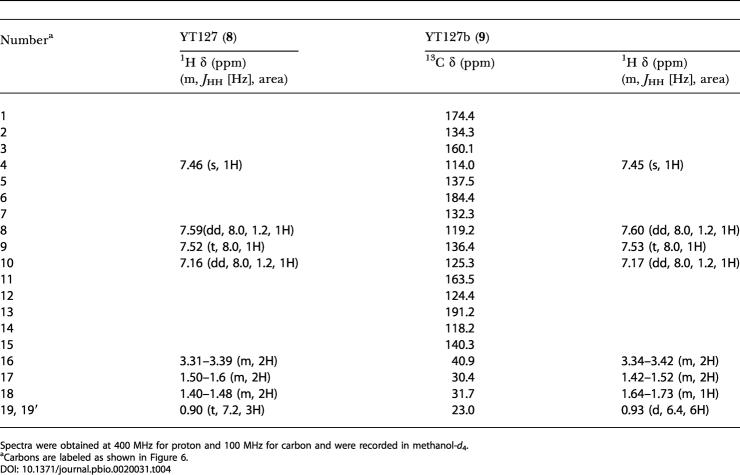
Proton and Carbon NMR Data for YT127 (**8**) and YT127b (**9**)

Spectra were obtained at 400 MHz for proton and 100 MHz for carbon and were recorded in methanol-*d*_4_

^a^Carbons are labeled as shown in Figure 6

### Cytotoxic Properties of Novel Anthraquinones

As described above, the biosynthetic engineering methods reported here have yielded practical routes for the production of several new as well as known anthraquinone compounds. Given the track record of this family of natural products as pharmacologically active molecules, compounds **3**,** 4**, **9**, and DMAC were assayed for cytotoxic activities against human mammary adenocarcinoma MCF-7 cells. Apoptosis was observed after 24 h of drug treatment, and IC_50_ values were recorded after 5 d of drug addition. The IC_50_ values for reduced compounds DMAC and **9 **are 26.9 and 21.7 μg/ml, respectively, while the IC_50_ values for unreduced anthraquinones **3 **and **4 **are 3.4 and 1.7 μg/ml, respectively. Thus inserting the hydroxyl group at C-9 results in a 10-fold increase in cytotoxic activity. The new compounds also show modest improvement in cytotoxic activity relative to the natural products **5 **(R1128b, IC_50_ = 9.5 μg/ml) and **6** (R1128c, IC_50_=6.2 μg/ml) (Hori et al. 1993), suggesting an additive effect of both the C-9 OH and C-2 COOH groups.

#### Inhibition of glucose-6-phosphatase.

**Figure 7:**
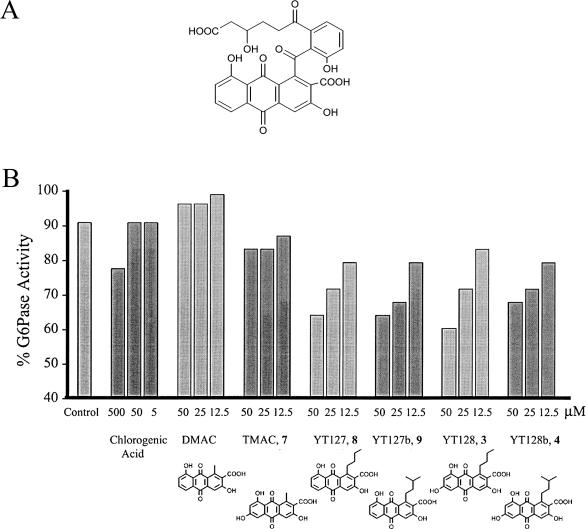
Inhibition of Glucose-6-Phosphate Translocase Activity by Anthraquinones (A) The chemical structure of mumbaistatin. Mumbaistatin is an extremely potent inhibitor of glucose-6-phosphate translocase (IC_50_ = 5 nM). The core of the molecule is a reduced carboxylic acid containing anthraquinone, which is also observed in compounds such as DMAC, YT127, and YT127b. (B) Inhibition of glucose-6-phosphate translocase activity by novel anthraquinones. The inhibition assay is performed as described in the experimental section. The control contains the rat liver microsome and glucose-6-phosphate only. Chlorogenic acid (IC_50_ = 0.26 mM) was used as a reference. The G6Pase activity of the microsome in the presence of six anthraquinones (DMAC, **3, 4, 7, 8**, and **9**) was measured. For each compound, three different concentrations (50, 25, and 12.5 mM) were used to detect dose-dependent inhibition. No inhibition was observed for DMAC or **7**. Dose-dependent inhibition was observed for compounds **3,** **4**,** 8**, and **9**.

Recently, the natural product mumbaistatin (Figure 7A) was identified as an extremely potent inhibitor (IC_50 _= 5 nM) of the glucose-6-phosphate translocase enzyme complex, an attractive target for the treatment of Type II diabetes (Vertesy et al. 2001). The core of mumbaistatin consists of an anthraquinone moiety that is related to several engineered compounds discussed in this report. For example, the carboxylic acid at position C-1 and the reduced C-9 are identical to those present in compounds DMAC, **8**, and **9**. We tested the inhibitory activities of some of these polyketides against glucose-6-phosphate translocase using intact male rat liver microsomes.

The integrity of the microsomes was first verified by comparing the glucose-6-phosphatase (G6Pase) activity in the presence of either glucose-6-phosphate or mannose-6-phosphate. The activity of G6Pase was then measured in the presence of chlorogenic acid (IC_50_ = 0.26 mM), DMAC, **3**,** 4**,** 7**,** 8**, or **9** using a colorimetric assay described earlier (Arion 1989) (Figure 7B). Dose-dependent inhibition was observed in the 10–50 μM range with the alkylacyl-primed compounds **3**,** 4**,** 8**, and **9**, but not DMAC or **7**. Our results demonstrate the following: (i) a long substituent at C-16 in an anthraquinone is important for targeting the membrane-bound G6Pase, and (ii) the C-9 position of the anthraquinone can be chemically modified without significantly affecting the enzyme-inhibitor interactions.

## Discussion

The engineering of the primer units of macrolide antibiotics is a well-established strategy for generating new natural product analogs with modified chemical and biological properties (Jacobsen et al. 1997; Marsden et al. 1998; Moore and Hertweck 2002). In contrast, manipulation of the ordinarily invariant acetate primer unit of bacterial aromatic polyketides has not been recognized as a general methodology in biosynthetic engineering, presumably owing to the apparently high efficiency with which these PKSs decarboxylate malonyl extender units to generate acetate primers. An exception to this principle has been recently demonstrated in the case of the enterocin PKS, which ordinarily incorporates a benzoic acid primer unit, but can also accept a range of aryl acids to generate substituted enterocins (Kalaitzis et al. 2003). In this report, we have described a general method for modifying the primer unit of any aromatic polyketide by engineering hybrid bimodular PKSs. This method can be used to construct hitherto undiscovered polyfunctional aromatic scaffolds, as illustrated by compounds **1 **and **2**; alternatively, regioselective modifications of known polyketides, such as **8 **and **9**, can be achieved. Notably, structural analysis of these novel compounds also revealed fundamentally new properties of bacterial aromatic PKSs, as summarized below.

### The KS-CLF Prefers Nonacetate Priming over Decarboxylative Priming

Most bacterial aromatic PKSs catalyze chain initiation by decarboxylating an ACP-bound malonyl extender unit to yield an acetyl primer unit, a reaction that is catalyzed by the KS-CLF. In order to install nonacetate primer units in an aromatic polyketide backbone, one must bypass this decarboxylative priming mechanism. Genetic and enzymological analysis of the R1128 PKS, which utilizes a range of nonacetate primer units, has revealed the existence of two PKS modules. Each module includes a distinct KS and an ACP. Previous studies have shown that these two KS-ACP pairs have orthogonal molecular recognition features, leading to the speculation that the initiation module may be able to productively interact with other bacterial aromatic PKSs to synthesize hybrid polyketides. However, the ability of the R1128 initiation module to kinetically compete with the intrinsic decarboxylative priming mechanism of the heterologous PKS was unexplored. To address this question, we coexpressed the entire R1128 initiation module with either the *act* or the *tcm* minimal PKS. The efficient biosynthesis of compounds described in this report shows that, although decarboxylation cannot be completely suppressed, both PKSs have an intrinsic preference for nonacetate primers over decarboxylative chain initiation. It should be noted that although acetate-primed products are observed in conjunction with nonacetate-primed compounds (e.g., CH999/pYT46 cosynthesizes mutactin along with compounds **1** and **2**), the former class of products may not be derived via decarboxylative priming in strains carrying bimodular PKSs. Instead, they may arise as a result of premature diketide transfer from the initiation module to the elongation module before the β-carbonyl can be reduced. Future isotope labeling studies on such systems should be useful for quantifying the distribution between polyketide chains derived from bimodular PKSs versus those that arise via decarboxylative priming.

Our findings are consistent with the fact that the frenolicin PKS from Streptomyces roseofulvus can synthesize both nanaomycin (an acetate-primed aromatic polyketide) and frenolicin (its butyrate-primed analog) (Tsuzuki et al. 1986). They also explain earlier observations that the doxorubicin (a propionate-primed polyketide) and oxytetracycline (a malonamate-primed polyketide) minimal PKSs yield acetate-primed polyketides, when expressed alone (Fu et al. 1994; Rajgarhia et al. 2001). Thus, notwithstanding its widespread prevalence, decarboxylative priming by the KS-CLF can be regarded as a default mechanism for chain initiation that occurs when alternative primer units are unavailable.

The potential for recombining naturally occurring initiation and elongation PKS modules from bacterial aromatic PKSs is enormous. Other than the R1128 biosynthetic pathway, initiation modules with attractive primer unit specificity can also be found in the doxorubicin, frenolicin, enterocin, and (presumably) oxytetracycline biosynthetic pathways. It should be possible to recombine these synthase units with elongation modules from the *act*, *frn*, *tcm*, and *whiE* (Yu and Hopwood 1995) PKSs (which synthesize C_16_–C_24_ backbones) to yield a range of reactive backbones whose subsequent fates can be controlled by previously analyzed auxiliary PKS subunits and tailoring enzymes.

### Molecular Recognition Features of the KS-CLF and Auxiliary PKS Subunits

By generating a variety of nascent and highly reactive alkylacyl polyketide intermediates in situ, we have been able to probe the properties of the key aromatic PKS components, including the KS-CLF, the KR, and different subclasses of AROs and CYCs. Such studies have provided insight as to whether carbon chain length of the backbone or the repetitive poly-β-ketone functionality are the primary factors influencing the substrate specificity of these proteins.

Our results demonstrate that, since chain length specificity of KS-CLF heterodimers is primarily dictated by the backbone size, incorporation of bulky, nonacetate primer units is compensated for by a reduced number of condensation cycles. Thus, hexaketides and octaketides are synthesized by the *act* and *tcm* KS-CLF, respectively, when these KSs are primed with pentanoyl (or 4-methylpentanoyl) primer units.

In contrast to KS-CLF subunits, the regioselectivity of auxiliary PKS enzymes such as KR, ARO, and CYC is unaffected by the incorporation of nonacetate starter units. For example, the *act* KR selectively reduced the C-9 keto group in an acetate-primed octaketide (DMAC), an alkyl-hexaketide (see Figure 4B), and an alkyl-octaketide (see Figure 5B). This observation confirms an earlier proposal (McDaniel et al. 1993b) that such KRs recognize fully synthesized polyketide chains, rather than the β-ketone of a partially elongated intermediate. Similarly, the *act* ARO and CYC process a reduced (but not unreduced) octaketide, regardless of the primer unit, although they are unable to recognize hexaketides or decaketides. In contrast, the R1128 CYCs act upon an unreduced, but not reduced, backbone, regardless of the primer unit.

### The Initiation Module of a Bimodular Aromatic PKS

Our studies have revealed that each initiation module component, ZhuG (see Figure 4A), ZhuH (data not shown), and ZhuC (data not shown) are essential for priming with a nonacetate building block. Deletion of any of these three genes from the constructs shown in Table 1 completely abolishes nonacetate priming by the KS-CLF, but leaves the decarboxylative priming mechanism intact. Although the roles of ZhuG and ZhuH in the initiation pathway have been reported (Meadows and Khosla 2001; Tang et al. 2003), the role of ZhuC is unclear. ZhuC is homologous to the MAT and was therefore putatively assigned as a second malonyl transferase that malonylates ZhuG. However, subsequently it was shown that MAT can malonylate ZhuG with high efficiency (*k_cat_*, approximately150 s^–1^) (Tang et al. 2003), whereas ZhuC is sluggish in malonylating ACPs (at a rate approximately ten times slower than MAT [data not shown]). In light of these observations, we propose that ZhuC catalyzes transacylation between the diketide-ZhuG and ZhuN, leading to an alkylacyl-ZhuN intermediate that can then be transferred onto the KS-CLF. Future biochemical analysis may be able to verify this property of ZhuC.

### Engineered Biosynthesis of Diverse Aromatic Polyketides via Bimodular PKSs

To further expand the repertoire of primer units that can be introduced into aromatic polyketides using bimodular PKSs, one could (i) alter the substrate specificity of the R1128 initiation module and/or (ii) engineer in vivo metabolic pathways for new types of primer units. KSIII homologs found in initiation modules serve as gatekeepers in primer unit selection and are therefore attractive targets for protein engineering. The X-ray crystal structure of ZhuH has recently been solved and has led to the identification of a binding pocket for the acyl-CoA moiety (Pan et al. 2002). ZhuH adopts a dimeric thiolase fold and selected residues at the interface between the two subunits control size and flexibility of the binding pocket, preventing acyl groups larger than isovaleryl groups from entering the pocket. The corresponding amino acids in FrnI, the homolog to ZhuH in the *frn* PKS, are occupied by bulkier residues, thus excluding acyl groups larger than acetyl-CoA. Therefore, altering the size and polarity of these gatekeeping residues using rational mutagenesis and directed evolution may enlarge the repertoire of acyl-CoA moieties recognized by KSIII enzymes.

Amino acid catabolism in S. coelicolor is the primary source of primer units such as isobutyryl-CoA (valine) and isovaleryl-CoA (leucine). These pathways involve transamination (catalyzed by branched-chain amino acid transaminases) to convert the amino acid into the corresponding α-ketoacid, followed by decarboxylation (catalyzed by acyl-CoA dehydrogenase [AcdH]) to yield the corresponding acyl-CoA (Zhang et al. 1999). These catabolic pathways in actinomyces are presumably tolerant of unnatural amino/α-keto acids, as illustrated by the incorporation of a large variety of primer units into the macrolide avermectin through precursor feeding (Dutton et al. 1991). It may therefore be possible to expand the repertoire of primer units in S. coelicolor by feeding the recombinant strains, constructed as above, with unnatural amino acids such as allylglycine, norvaline, norleucine, and fluorinated derivatives thereof. In addition, heterologous expression of enzymes involved in the biosynthesis of novel acyl-CoA moieties, such as cyclohexynoyl-CoA (Cropp et al. 2000) and benzoyl-CoA (Xiang and Moore 2003), can be efficient sources of loading module substrates. Successful elaboration of the corresponding acyl-CoA primers into full-length polyketides, through both KSIII-based protein engineering and *Streptomyces* metabolic engineering, can yield additional polyketide variants. Our observation that anthracycline derivatives generated via such engineering approaches can have improved properties over their natural product counterparts provides further motivation to expand the biosynthetic potential of bimodular PKSs.

## Materials and Methods

### 

#### Bacterial strains and general methods for DNA manipulation.

S. coelicolor strain CH999 was used as the host for transformation by shuttle vectors. Protoplast preparation and PEG-assisted transformation were performed as described by Hopwood et al. (1985). All cloning steps were performed in Escherichia coli strain XL-1 Blue. PCR was performed using the *pfuTurbo* polymerase (Strategene, La Jolla, California, United States). PCR products were first cloned into pCRBlunt vector (Invitrogen, Carlsbad, California, United States), followed by DNA sequencing (Stanford PAN Facility, Stanford, California, United States). Unmethylated DNA was obtained by using the methylase-deficient strain GM2163 (New England Biolabs, Beverly, Massachusetts, United States).

#### Construction of plasmids.

The following primers were used to amplify the individual genes: *zhuC*: 5′-CC*TCTAGA*TGTACTCGGGTCGAGGAGACCTCCG-3′, 5′-GG*ACTAGT*GCCACGTTCACCGTTCCGCCGCG-3′; *zhuN*: 5′-CATGCGACCCG*TCTAGA*GAAGGAGATTCCG-3′, 5′-CGCGGTTCTGC*ACTAGT*CAGGCCGCGGCC-3′; *zhuG*: 5′-CCTG*TCTAG*AGGGAGGACGAACCC-3′, 5′-TG*CTGCAG*TCAGCCCGCGGTCTCG-3′; *zhuH*: 5′-GA*CTGCAG*CAGAACCGCGAAAGGTGG-3′, 5′-AGTAGTAC*GTTTAAAC*TCAAGCCGGAGTGGACGGC-3′; *act*VII*/act*IV: 5′-GCC*GTTTAAAC*GCTGGCGCCAAGCTTCTC-3′, 5′-CCGGAGACGCGTCACGGCCGAAGC-3′; *zhuI/zhuJ*: 5′-GCC*GTTTAAAC*CGAGGAGCACCCTCATGCGTC-3′, 5′-GGACTAGTCCTCCTCTTCCTGCTCG-3′. The introduced restriction sites are shown in italics. Genes encoding *zhuC*, *zhuH*, *zhuN*, *zhuG*, and *zhuI/zhuJ* were amplified from pHU235 (Marti et al. 2000), and genes encoding *act*VII*/act*IV were amplified from pRM5 (McDaniel et al. 1993a). *zhuC*, *zhuN*, and *zhuG* were cloned as a single 2.1 kb XbaI–PstI cassette; *zhuH* was cloned as a 1.3 kb PstI–PmeI cassette; *act*VII*/act*IV (2.5 kb) and *zhuI/zhuJ* (1.3 kb) were each cloned as a PmeI–EcoRI cassette. Different combinations of cassettes, as shown in Table 1, were introduced into either pRM5 (KR-*act*KS/CLF), pSEK24 (*act*KS/CLF), pSEK33 (*tcm*KS-CLF), or pRM20 (KR-*tcm*KS/CLF) to yield pYT46, pYT105, pYT128, and pYT127, respectively.

#### Culture conditions, extraction, and small-scale analysis.

The strains were grown on R5 plates containing 50 mg/l thiostrepton at 30°C for 7–10 d. Acyl-CoA precursors such as sodium propionate and valine were added at 1 g/l when needed. For LC/MS and analytic HPLC analysis, one plate was sufficient. The plate was chopped into fine pieces and extracted with 30 ml of ethyl acetate (EA)/methanol/acetic acid (89:10:1). The solvent was removed in vacuo and the residue was redissolved in 1 ml of methanol. The polyketide products were separated and detected by analytical reversed-phase HPLC using a diode array detector at 280 and 410 nm using an Alltech (Vienna, Virginia, United States) Econosphere C18 column (50 mm × 4.6 mm); linear gradient: 20% acetonitrile (ACN) in water (0.1% TFA) to 60% ACN in water (0.1% TFA) over 30 min; 1 ml/min. HPLC retention times (t_R_, minutes) were as follows: **1**: 13.8; **2**: 15.4; **3**: 20.4; **4**: 21.8; **5**: 24.9; **6**: 26.4; **7**: 17.1; **8**: 22.8; **9**: 24.4; **10**: 27.7;** 11**: 29.4. LC/MS was performed at the Vincent Coates Foundation Mass Spectrometry Laboratory at Stanford University using a ThermoFinnigan (San Jose, California, United States) quadrupole ion trap LC/MS system and electrospray ionization (both positive and negative ionization).

#### Large-scale production and isolation.

Sufficient number of R5 plates (20–60 plates, depending on the yield of the product) streaked with the desired CH999 strains were grown at 30°C for 1 wk. The plates were chopped into fine pieces and extracted with a minimum of 1 l of EA/methanol/acetic acid (89:10:1). The organic solvents were removed and the residuals were dissolved in 5 ml of methanol. The solution was filtered and injected into a preparative reversed-phase HPLC column (250 × 22.5 mm C-18 column; Alltech Econosil). A 20%–60% ACN in water (0.1 % TFA) gradient (50 min, 5 ml/min) was used to separate the polyketide products. Fractions containing the desired polyketides were combined and concentrated in vacuo. The residuals were redissolved in acetone and applied to a preparative TLC plate (20 cm × 20 cm, 0.25 mm E. Merck [Readington Township, New Jersey, United States] silica gel plates [60F-254]). TLC plates spotted with **1** (*R*_f_ = 0.34) and **2** (*R*_f_ = 0.41) were developed with EA/methanol/acetic acid (97:2:1), while those spotted with **3 **(*R*_f_ = 0.29), **4 **(*R*_f_ = 0.37), **7** (*R*_f_ = 0.21), **8 **(*R*_f_ = 0.34), and **9** (*R*_f_ = 0.43) were developed with EA/hexane/acetic acid (90:10:1). The desired bands were excised from the TLC plates and stirred in EA/methanol (10 ml, 9:1) for 2 h. The compounds were eluted from silica using the same solvent and dried in vacuo.

#### NMR and MS characterization of novel compounds.

NMR spectra were recorded on Varian (Salt Lake City, Utah, United States) Inova 500 or Mercury 400 instruments and calibrated using residual undeuterated solvent as an internal reference.^1^H and ^13^C NMR spectra data are shown in Tables 2–4. HRFABMS were collected under negative ionization mode as follows: HRFABMS m/z: **1**: 277.1082 (calcd for C_15_H_17_O_5_: 277.1076); **2**: 291.1247 (calcd for C_16_H_19_O_5_: 291.1232);** 3**: 355.0823 (calcd for C_19_H_15_O_7_: 355.0818); **4**: 369.0981 (calcd for C_20_H_17_O_7_: 369.0974); **7**: 313.0354 (calcd for C_16_H_9_O_7_: 313.0348); **8**: 339.0871 (calcd for C_19_H_15_O_6_: 339.0869); **9**: 353.1036 (calcd for C_20_H_17_O_6_: 353.1025).

#### Cytotoxicity studies.

The studies were performed as described by Hori et al (1993). The cells were maintained at 37°C and growth was measured with the colorimetric MTT assay after each day. The IC_50_ values were measured after 5 d.

#### G6Pase activity assay.

Male rat liver microsome (Sprague Dawley) was purchased from BD Gentest^TM^ (Becton Dickinson, Franklin Lakes, New Jersey, United States). Aliquots (100 μl, 2 mg/ml) in 0.25 M sucrose were stored at –80°C. Compound stock solutions were prepared in 95% ethanol and diluted with DMSO. Glucose-6-phosphate and mannose-6-phosphate were purchased from Sigma (St. Louis, Missouri, United States).

The integrity of microsomes and G6Pase activity was measured based on the colorimetric reaction of inorganic phosphate as previously reported (Arion 1989). The enzyme reaction was initiated by adding 3 μl of microsome to the reaction mixture, which contained 51 μl of assay buffer (50 mM HEPES, 100 mM KCl, 2.5 mM EDTA, 2.5 mM MgCl_2_, and 1 mM DTT at pH 7.2), 3 μl of glucose-6-phosphate (final concentration, 1 mM), and 3 μl of inhibitor sample in DMSO. The reaction mixture was incubated at room temperature, and 13 μl of reaction mixture was taken every 10 min and quenched with 117 μl of working solution (6:2:1 mixture of 0.42% ammonium molybdate tetrahydrate in 1N H_2_SO_4_, 10% SDS in water, and 10% ascorbic acid in water). The blue reduced phosphomolybdate complex is formed after incubation at 50°C for 20 min. The absorbance was measured at 820 nm.

## Supporting Information

### Accession Numbers

The SwissProt (www.ebi.ac.uk/swissprot/) accession numbers for the proteins and genes discussed in this paper are *act* ARO/CYC (Q02055), *act* KS-CLF (Q02059 and Q02062), ZhuA (Q9F6E1), ZhuB (Q9F6E0), ZhuC (Q9F6D6), ZhuG (Q9F6D5), ZhuH (Q9F6D4), ZhuI (Q9F6D3), ZhuJ (Q9F6D2), and ZhuN (Q9F6C8).

## Abbreviations

AcdH: acyl-CoA dehydrogenase
ACN: acetonitrile
ACP: acyl carrier protein
*act*: actinorhodin
ARO: aromatase
AT: acyl transferase
CLF: chain length factor
CYC: cyclase
DMAC: 3,8-dihydroxy-methylanthraquinone carboxylic acid
EA: ethyl acetate
FAS: fatty acid synthase
G6Pase: glucose-6-phosphatase
KR: ketoreductase
KS: ketosynthase
LC: liquid chromatography
MAT: malonyl-CoA:ACP acyl transferase
MS: mass spectrometry
NMR: nuclear magnetic resonance
PKS: polyketide synthase
*tcm*: tetracenomycin
TMAC: 3,6,8-trihydroxy-1-methylanthraquinone-2-carboxylic acid
